# Experimental Study of Used Wind Turbine Blades for Their Reuse in Slope and Trench Protection

**DOI:** 10.3390/ma17194934

**Published:** 2024-10-09

**Authors:** Lidia Buda-Ożóg, Anna Halicka, Mirosław Broniewicz, Joanna Zięba, Damian Nykiel, Łukasz Jabłoński, Filip Broniewicz

**Affiliations:** 1 Department of Building Structures, Faculty of Civil Engineering and Environmental Engineering, Rzeszow University of Technology, Poznańska 2, 35-084 Rzeszów, Poland; j.zieba@prz.edu.pl (J.Z.); d.nykiel@prz.edu.pl (D.N.); 2 Department of Building Structures, Faculty of Civil Engineering and Architecture, Lublin University of Technology, Nadbystrzycka 40, 20-618 Lublin, Poland; a.halicka@pollub.pl (A.H.); l.jablonski@pollub.pl (Ł.J.); 3 Department of Building Structures and Structural Mechanics, Faculty of Civil Engineering and Environmental Sciences, Bialystok University of Technology, Wiejska 45A, 15-351 Bialystok, Poland; m.broniewicz@pb.edu.pl (M.B.); filip.broniewicz@pb.edu.pl (F.B.)

**Keywords:** wind turbine blades, repurposing, retaining wall, distributed fiber optic sensing, digital image correlation, strength properties, strains, displacements

## Abstract

This article presents the results of an experimental study carried out to assess the possibility of using waste wind turbine blades as retaining wall structures for slopes and trenches. The use of Vestas and LM-type blades as retaining wall components was assumed, based on ‘columns’ made of Vestas-type closed profiles filled with concrete and ‘slabs’ of fragments extracted from LM-type blades. The results of the tests and comparisons of the displacement and strain values of the components obtained using different measurement methods are presented in this paper. The force–strain and force–displacement relationships obtained from the tests were used to validate numerical models of slope protection walls and excavations designed from used wind turbine blades. According to our research, there is a high degree of variability in the strength parameters and deformation of the composite elements made from the wind turbine blades. Therefore, in the case of this type of material, characterized by a significant variation in carrying capacity, deformability, and the nature of the failures, the use of different measurement methods makes it possible to obtain much of the data necessary for assessing the reusability of wind turbine blades in building.

## 1. Introduction

The need to protect the environment and energy security are the reasons renewable energy sources are becoming increasingly important. Wind turbines are one of the most popular green energy sources that have been used worldwide for years, which are made of composite materials and designed only for a service life of 20–25 years. Once the blades have reached their end of life (EOL), they are decommissioned regardless of their condition.

It is difficult to accurately estimate the number of end-of-life wind turbine blades that will be recycled in the coming years. During the past 15 years, several projections of the amount of future wind turbine blade waste have been proposed and reported in the scientific literature. For example, the ETIPWind Executive Committee predicts that in Europe, the amount of end-of-life wind turbine blades should be greater than 66 tons by 2025 [1]. However, in the longer term, Barlow [2] estimates that the total mass of wind turbine blade waste could be as high as 2 MT by 2050. The regional distribution indicates that China will be responsible for most of the waste (40%), followed by Europe (25%), the rest of the world (19%), and the USA (16%). As indicated by the large number of wind turbine blades found in landfill sites, the timing suggests that Europe will be the first to deal with this issue [3].

The materials used in wind turbine blades are generally composed of the following:

- Reinforcing fibers—usually glass—although they can also be carbon, aramid, or basalt;
- Polymer matrix, e.g., thermosets such as epoxies, polyesters, vinyl, esters, polyurethane (PUR), or thermoplastics;
- Sandwich core, e.g., balsa wood or foams, e.g., polyethylene terephthalate (PET);
- Coatings, e.g., polyethylene (PE) or PUR;
- Metals, e.g., copper wiring and steel bolts.

Although material compositions vary between blade types and blade manufacturers, typically about 80% of the weight is glass fiber-reinforced thermoset polymer composites, which are technically difficult to recycle and convert into new materials. This means that proper disposal of the fragments of wind turbine blades is one of the most challenging aspects due to the size of the components, the complexity of recycling, and the low market value. Furthermore, the storage or incineration of wind turbine blades is extremely harmful, and these activities can result in serious health problems and environmental impacts [4]. Current initiatives and research on wind turbine blade reuse are multifaceted and include reuse, repurposing, recycling, reclamation, coprocessing, incineration, landfill, and storage [5,6,7,8,9,10]. Each of these methods must be evaluated with respect to their overall sustainability, including economic feasibility, environmental impact, and social acceptance [11]. As the composite material of wind turbine blades is strong, lightweight, and durable, it can be used effectively in various areas of the economy, creating new components with a relatively high value.

To address the global problem of high volumes of wind turbine blade recalls, a ‘Re-Wind Network’ has been established—a consortium of several universities such as Queen’s University Belfast, University College Cork, Georgia Institute of Technology, and City University of New York. The main objective of the research is to assess the feasibility of using end-of-life wind turbine blades in a second project [12]. In 2017, the team began the investigation by identifying wind blades for use in a pedestrian bridge demonstration project. In addition to work related to the use of blades in bridges, the team investigated the use of blades as poles for power transmission. The Re-Wind Network has also proposed numerous re-purposing solutions, which can be found in the Re-Wind Fall 2022 project catalogue [13]. Other countries in Europe and organizations such as ReBlade, Blade-Made, and Anmet [14] have also taken initiatives to repurpose wind turbine blades.

However, achieving suitable design applications for wind turbine blades in construction is not a simple task. Before application, the wind turbine blade waste or its fragments require a thorough assessment of the damage condition and material properties due to operation under different conditions and at different times. Furthermore, from a design point of view, wind turbine blades have variable structures, thickness, and shapes, so it is difficult to manufacture uniform components, and ensuring consistent and unchanging material parameters is almost impossible. Therefore, extensive material and strength testing, as well as the development of suitable material models required for computational analyses, must be performed before the efficient use of wind turbine blade fragments in construction.

The concept of repurposing wind turbine blade waste for slopes and trenches as retaining walls is the subject of this article. These can be ad hoc protection, e.g., for the duration of construction works, or permanent protection related to a non-uniform terrain level. In the longer term, due to the location of a significant number of wind farms offshore, the analyzed solution may be used for strengthening shorelines and cliffs. The use of wind turbine blades as strengthening elements requires the determination of their strength parameters. Although it is relatively easy to determine the tensile and compressive strengths of individual composite specimens, determining the load-bearing capacity of an entire structure dedicated to protecting an excavation or slope of the earth is a significant challenge. An alternative solution may be to develop and experimentally verify numerical models of the designed protection systems.

This article presents material and structural testing of individual components obtained from waste blades from LM and Vestas wind turbines. The results obtained, particularly the force–displacement and force–deflection relationships, will be used to validate preliminary numerical models for the components under investigation. The FE models prepared in this way will form the basis for the construction and validation of the final FEM model of the slope protection and excavation designed.

## 2. The Subject of Analyses and Measuring Methods Applied to It Components

### 2.1. Retaining Wall Concept

**Figure 1:**
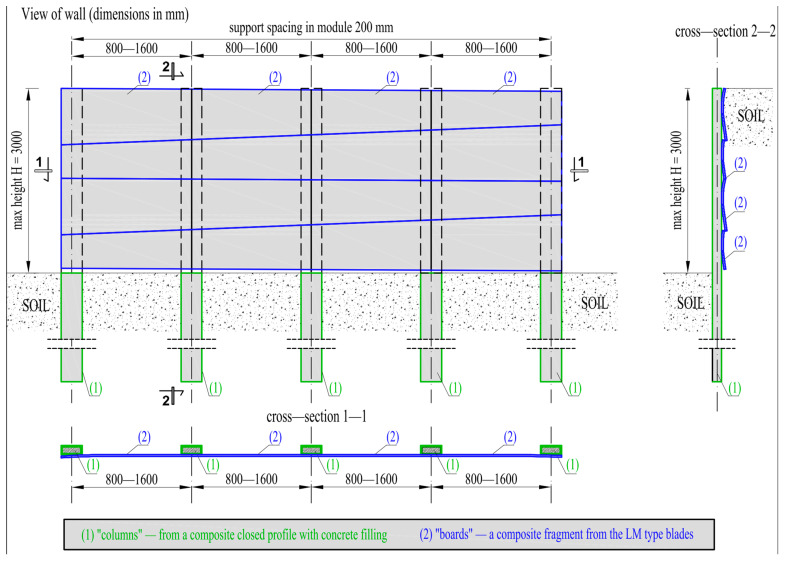
Construction scheme of the retaining wall.

The concept of repurposing wind turbine blade waste for slopes and trench protection walls involves the use of two types of elements (Figure 1). These are vertical columnar elements obtained from Vestas-type blades and horizontal plate elements obtained from LM-type blades. The limited height of the wall above ground level is 3000 mm. The minimum foundation depth of the columns is determined by the stability and load-bearing capacity conditions and depends on soil conditions. The spacing of the supports is designed in the range of 800–1600 mm in a 200 mm module. Columns are designed from a closed composite profile obtained from Vestas-type blades. Columns with a maximum length of up to 6 m are inserted into the soil at least to the height of the wall, depending on soil conditions. The columns are designed with concrete filling. The boards are designed from the side walls of Vestas-type blades. The cross-sections of the boards have a slightly curved surface, obtained as wing fragments with dimensions of about 500 × (950–1750) mm.

The boards are fastened to the columns alternately due to the variable geometry along their length. This ensures the security of the wall.

Cantilevered retaining walls can be used in trench support systems in congested areas for temporary and permanent structures to retain backfill material along with additional pressure in the form of buildings, vehicles, roadways, etc. [15]. The holding height is limited because a cantilever sheet pile wall derives its stability mainly from contact stresses developed below the dredge level along the embedded depth of the wall [16]. There are two failure modes to consider when designing a wall: first, parts of the wall may not be strong enough to resist the acting forces and, secondly, the wall as a rigid body can be displaced or overturned by the earth pressure acting on it. However, it is not possible to properly design a retaining wall, taking into account all the possible loads and failure mechanisms, without knowing the strength and deformability of the individual components. Therefore, a reliable assessment of these parameters must be the first step in the concept of repurposing wind turbine blade waste for slope and trench protection walls.

### 2.2. Measuring Methods

Various types of measurement methods are used to measure strains and displacements in experimental studies. Methods used in the testing of the composite fragment from LM/Vestas-type blades described later in this article include the following: distributed fiber optic sensing (DFOS), linear variable differential transformers (LVDTs), resistance foil strain gauges (RFSGs), and the ARAMIS 2D digital image correlation (DIC) system. The use of strain gauges and inductive sensors is well known, and they have been used in research for many years.

The DFOS fiber optic measurement technique makes it possible to achieve measurements of selected physical quantities with a spatial resolution so low compared to the total measurement length that they can be treated as geometrically continuous from an engineering point of view [17]. DFOS is a type of measuring tool that, in addition to interferometric sensors and sensors with a Bragg grating, is increasingly used in the monitoring of various structures [18]. Fiber optic measurement technology enables quasi-continuous measurements, as it allows several measurement points to be placed along a single fiber. From an engineering point of view, the individual measurements obtained in this way, i.e., from sensors spaced between 5 and 10 mm apart, can be considered continuous [19]. For example, over a length of 1 m, it is possible to obtain information about the strain’s value even at 200 measurement points. Basic DFOS measurement equipment includes optical fibers, monolithic fiber optic sensors, and layered sensing cables. The main physical quantity measured with DFOS is the strain [με]. In the presented experimental tests, a 9/125 standard single-mode fiber in its primary coating (external diameter equal to 250 µm) was selected. The technical specifications of this 9/125 standard single-mode fiber are summarized in the literature [17]. The fiber optic signal receiving station requires a computer with the appropriate software and a recorder—the LUNA OBR (Optical Backscatter Reflectometer).

Methods that enable the analysis of changes in the state of displacement and strain in larger areas are accepted as field methods. Among the field analysis methods, optical methods are the most numerous and widely used [17,20]. The DIC method consists of taking a series of images of the analyzed area of the studied structure at different loads and then analyzing the taken images to calculate the displacements of selected points of the analyzed area. In the research conducted, the ARAMIS 2D DIC system was used with a single 12 MPx camera (4096 × 3000 pixels). It consists of a set of cameras that record changes in the shape of the object under study and a suitably adapted and programmed computer that stores and processes the recorded images. The ARAMIS system measurements are based on the principle of digital image correlation. They allow material-independent and contact-free measurements of displacements and strains on surfaces. In the experimental tests presented, the GOM system was used, which includes a camera with two LED lamps and a computer with GOM software. The use of ARAMIS requires special preparation of the measured surface or markers.

## 3. Laboratory Research

### 3.1. Composite Components Under Test

**Figure 2:**
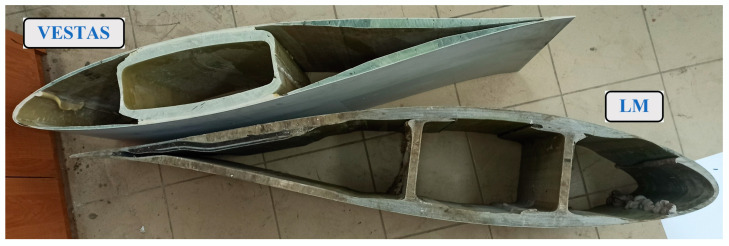
Cross-section of wind turbine blades.

Assessing the possibility of using wind turbine blade fragments for slope and trench retaining is not possible without carrying out material tests and strength tests on real-scale components. Despite the many tests carried out [21,22,23,24,25], due to the complex nature of the material (laminate) and the variability of its parameters over time, detailed test results are not available for the wind turbine blades considered for use. The variability of the cross-section of the composite elements and their material characteristics determine the need to perform tests on real-scale elements, with additional consideration of the planned joint with concrete using a composite closed profile obtained from the Vestas-type blades. The elements tested experimentally were horizontal composite elements and vertical composite elements, referred to in working terms as “boards” and “columns”. Components were extracted from the wind turbine blades in the cutting process (Figure 2).

**Figure 3:**
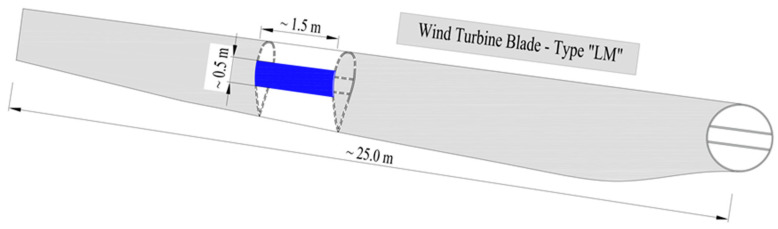
Scheme of LM-type wind turbine blade sampling: boards.

**Figure 4:**
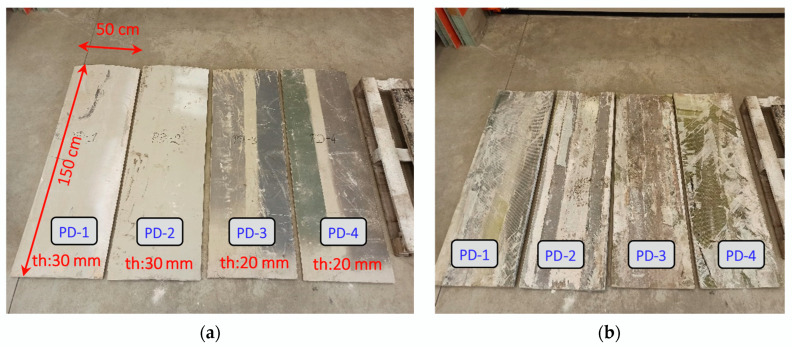
Prepared samples of “boards” from wind turbine blades of type LM: PD-1, PD-2, PD-3, and PD-4: (**a**) outer side of wind turbine blade; (**b**) inner side of wind turbine blade.

**Figure 5:**
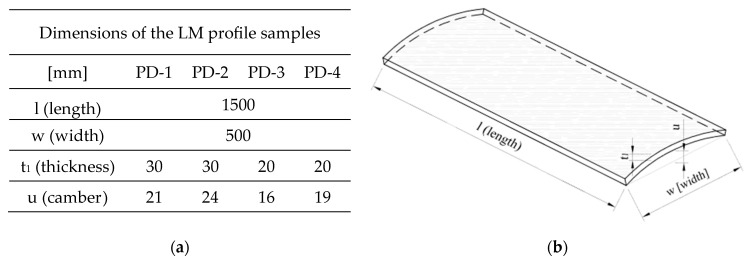
(**a**) Dimensions of the LM profile samples; (**b**) dimension marking.

A scheme for sampling the blades of an LM-type wind turbine is shown in Figure 3 below. Two types of board were analyzed, with fixed lengths of 150 cm, a width of 50 cm, and thicknesses of 30 and 20 mm. Four samples were prepared which differed in the thickness of the composite wall and were named PD-1, PD-2, PD-3, and PD-4. A view of the prepared samples of LM-type wind turbine blades is shown in Figure 4 and their dimensions are given in Figure 5.

**Figure 6:**
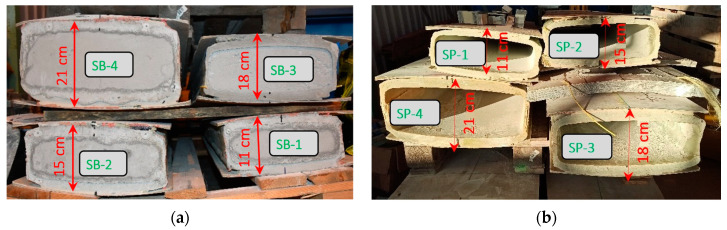
Samples prepared from wind turbine blades of the Vestas type: (**a**) filled with concrete; (**b**) not filled with concrete.

Fragments with a length of approximately 150 cm and a distinct height section were also taken into consideration in the closed profile derived from the Vestas-type blades. Figure 6 shows the four closed profiles that were prepared for testing: four filled with concrete (SB-1, SB-2, SB-3, and SB-4) and four empty (SP-1, SP-2, SP-3, and SP-4).

**Figure 7:**
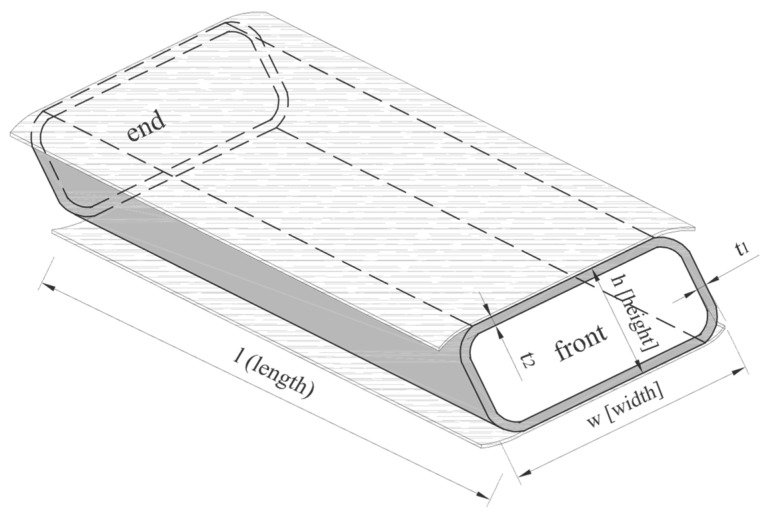
Dimension markings on Vestas profile samples.

In Figure 7, the box section test elements were characterized by variations in dimensions due to variations in the shape of the wind turbine blades. Their detailed dimensions are presented in Table 1.

### 3.2. Concrete and Composite Material Tests

Samples were taken from the tested composite elements to determine the tensile strength of the material. Tests were carried out on 14 samples taken from LM-type wind turbine blades (boards) and 14 samples taken from hollow sections of Vestas-type wind turbine blades. The samples were prepared in accordance with the ASTM D3039/D3039M standard and tested in a classic tensile test at a rate of 5 mm/min.

In the case of LM profile samples, the average longitudinal tensile strength is 401 MPa and the standard deviation is 37.5 MPa. The results obtained are similar to the mechanical properties related in the literature [26,27,28,29].

**Figure 8:**
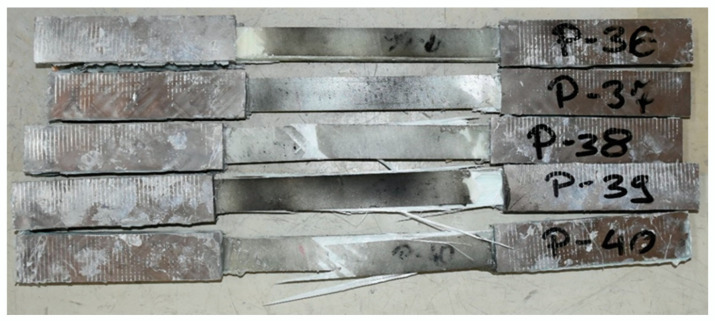
View of Vestas-type blade samples after destruction.

In the case of samples with a Vestas profile, the longitudinal tensile strength was characterized by high variability. Eight specimens, between 23 and 26 mm thick, taken from the top or bottom surfaces of the boxes failed within the strength machine mounts, while only one failed correctly in the center. The samples taken from the sides of the boxes, between 7 mm and 11 mm thick, all failed as recommended by the ASTM D3039/D3039M standard, shown in the photograph in Figure 8. Based on the Dixon test from the population surveyed, three outliers were rejected. The average longitudinal tensile strength of the Vestas-type elements obtained from the tests was only 297 MPa, with a standard deviation of 52.9 MPa.

Four Vestas profiles (SB-1, SB-2, SB-3, and SB-4) were filled with concrete of the strength parameters shown in Table 2.

The strength parameters of the concrete used to fill the box sections extracted from Vestas wind turbine blades, shown in Table 2, are characterized by a very low dispersion coefficient.

### 3.3. Monitoring System and Course of the Study

**Figure 9:**
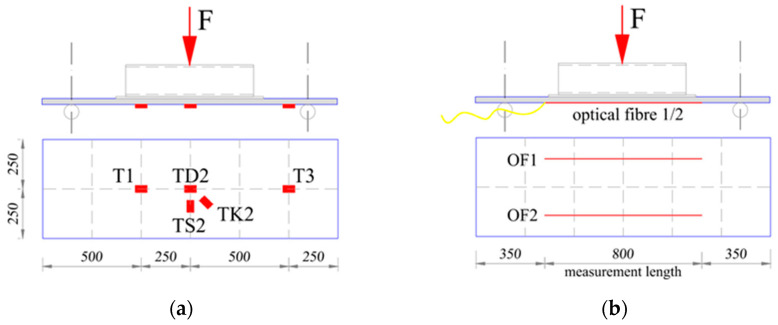
Distribution of RFSGs and optical fibers on LM profile samples PD-1, PD-2, PD-3, and PD-4: (**a**) scheme of distribution of RFSGs; (**b**) scheme of distribution of optical fibers.

**Figure 10:**
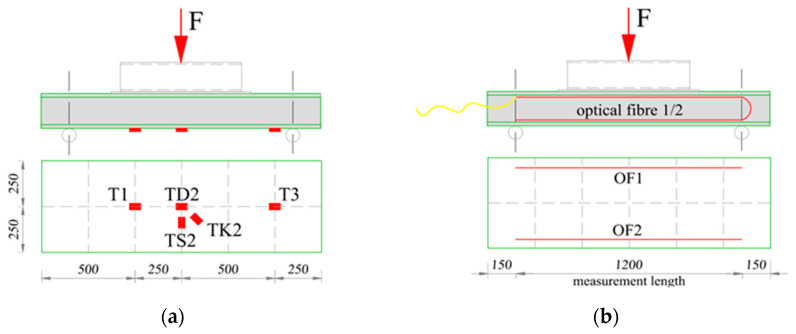
Distribution of RFSGs and optical fibers on Vestas profile samples: (**a**) scheme of distribution of RFSGs; (**b**) scheme of distribution of optical fibers.

The individual elements of the designed retaining wall that were the subject of the experimental tests, i.e., boards and columns, were tested for bending in a horizontal position. The board elements on the test bench were always arranged in the same way, i.e., with a downward curve. During the experimental tests, force, deflections, and strains were measured. Resistance foil strain gauges and distributed sensing fibers were used to measure strains. Both pieces of measuring equipment were installed on the surface of the bottom board (Figure 9). In the case of hollow sections, which would be used as a column in the retaining wall, strain gauges made of resistance film were installed on the bottom surface and diffuse sensor fibers were installed on the side surface (Figure 10).

**Figure 11:**
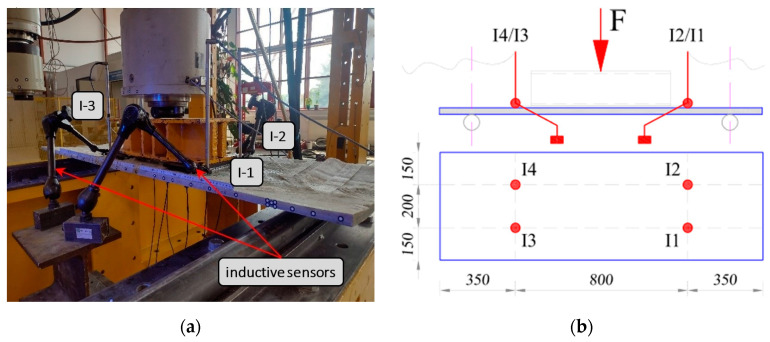
Distribution of inductive sensors: (**a**) photo of the sample; (**b**) scheme of distribution.

Four inductive sensors were used to measure vertical displacement. Their locations are shown in Figure 11; this scheme of sensor positions was used in the testing of all LM and Vestas samples. To avoid damaging the sensors due to the fragile failure of the composite materials tested, it was decided to remove them at an early stage of the test.

**Figure 12:**
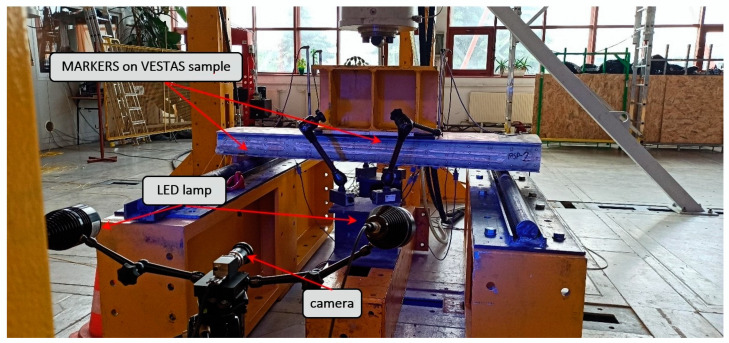
View of the Vestas profile sample ready for DIC measurements by the ARAMIS 2D system.

The use of the DIC ARAMIS 2D system to measure displacements required the appropriate preparation of the measured surface. The lateral surface of the test components was measured, which was clearly visible to the cameras of the ARAMIS system. On these surfaces, sticker markers were also used as measuring points (Figure 12).

The elements were loaded with a static scheme similar to four-point bending. However, due to the susceptibility of the composite to failure by punching, the load was applied uniformly over a significant contact area (Figure 11 and Figure 12).

During the experimental tests, force, deflections, and strains were measured. All research was carried out in the laboratory of the Faculty of Civil Engineering, Environmental Engineering, and Architecture at Rzeszow University of Technology (Poland).

## 4. Results and Discussion

In this section, the results of the selected tests are presented. The examples were selected in such a way as to show the versatile capabilities of all different measurement techniques: foil strain gauges, distributed sensing fibers, inductive sensors, and the ARAMIS digital image correlation system.

### 4.1. Measurement Results of Components PD-1, PD-2, PD-3, and PD-4—“Boards”

**Figure 13:**
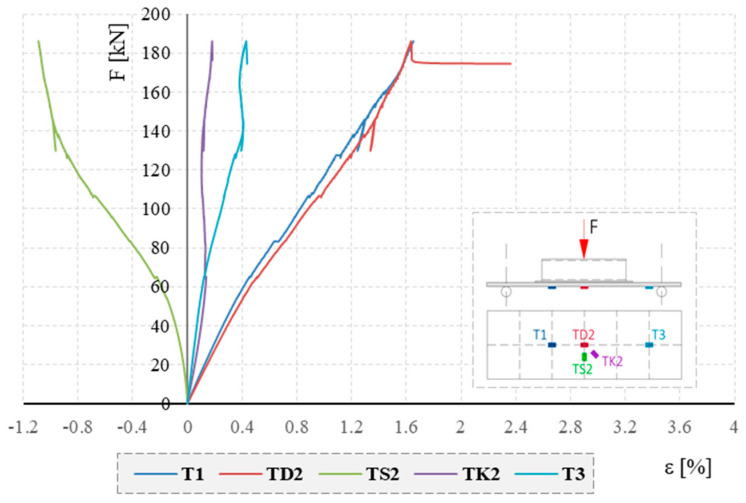
Strain–force relationship obtained from RFSGs for the PD-1 element.

The strain–force relationship obtained from the resistance foil strain gauges (RFSGs) for the selected board (PD-1) is shown in Figure 13.

The highest strain values were obtained for foil strain gauges placed at the mid-span of the element and measuring longitudinal strain, i.e., T1 and TD2. Negative transverse strains measured with the TS2 strain gauge indicate localized deformation of the bottom surface of the tested boards. This is because the applied load was not distributed over the entire width of the element and the initial shape of the element (camber downwards). Analogous strain–force relationships were obtained for the other tested board elements. To compare the results obtained, the selected strain values for all tested boards are summarized in Table 3. The maximum strain observed at 186 kN load (element PD-2) was equal to 1.78%.

**Figure 14:**
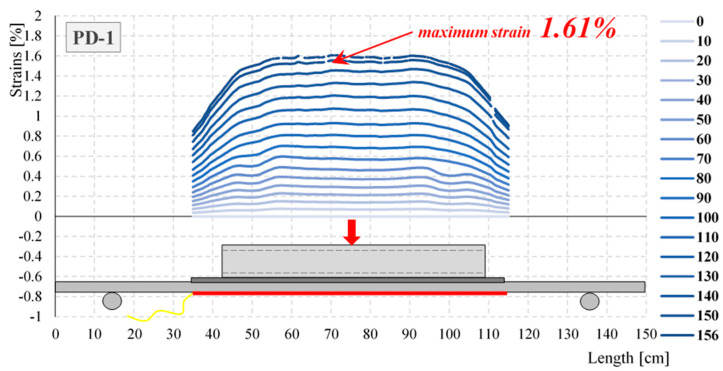
Strain distributions measured by DFOS fiber for the PD-1 element.

Figure 14 shows the strain distributions registered during the selected stage of the test for element PD-1, obtained from the optical fiber (OF). Extreme values were registered in the mid-span of the element. The maximum strain observed at a load of 156 kN was equal to 1.61% (16 100 με). The maximum strains of the elements PD-1, PD-2, PD-3, and PD-4, measured by the optical fibers (OF) for selected load stages, are summarized in Table 3.

Comparing the results in Table 3, it can be seen that in each case, the strains measured at each load value are lower than those measured with the fiber optic sensors. However, the maximum differences in the results obtained do not exceed 20% and, in the case of maximum strain values, 11%. The locations of the measurement sensors in the two measurement techniques, i.e., the different distances of the strain gauges and optical fibers from the axis of the board and its camber, can influence the differences in the results obtained.

**Figure 15:**
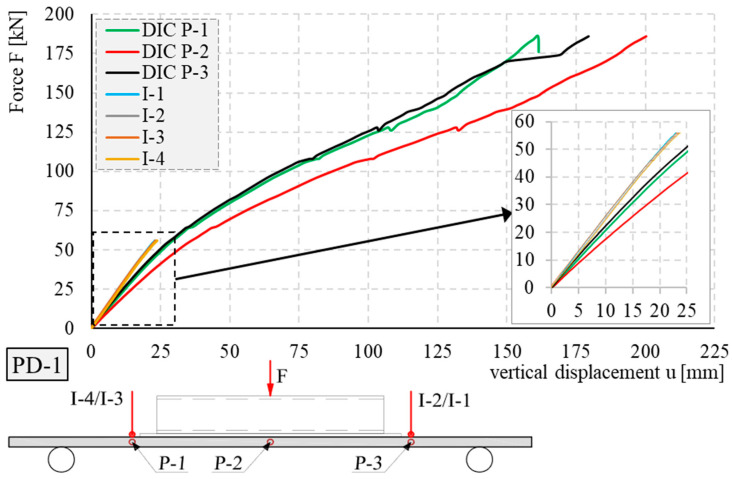
Displacements of the tested element PD-1.

The displacement–force relationship for the PD-1 element obtained during the tests is shown in Figure 15. Displacements were measured using inductive sensors and the DIC system. When the pre-estimated displacements were exceeded, the inductive sensors were removed because of the possibility of damage to the measuring sensors by separating composite fibers.

The maximum displacements of the elements PD-1, PD-2, PD-3, and PD-4, measured by the DIC system for selected load steps and selected markers P-1, P-2, and P-3, are summarized in Table 4.

**Figure 16:**
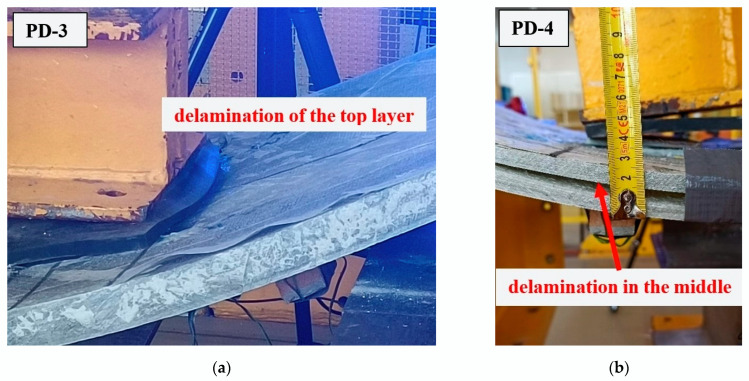
Failure mechanism of the components: (**a**) PD-3; (**b**) PD-4.

Both 30 mm thick boards (PD-1 and PD-2) showed significant agreement between the results obtained, i.e., the failure load, measured strains, and displacements. The situation was different for the 20 mm thick boards. The failure load for the PD-3 component was more than double that of PD-4. This phenomenon is also evident in the measured strains and displacements. The reason for this may be the different failure mechanisms of the PD-3 and PD-4 components, shown in Figure 16. In the PD-4 element, delamination occurred in the middle of the height, while in the PD-3 element, delamination occurred on the outer layer of the composite. The failure mechanism of the PD-1 and PD-2 boards was analogous to that of PD-3, i.e., due to delamination of the top layer of the element in the compression zone.

### 4.2. Measurement Results of Components SP and SB—Empty and Concrete-Filled Closed Profiles

**Figure 17:**
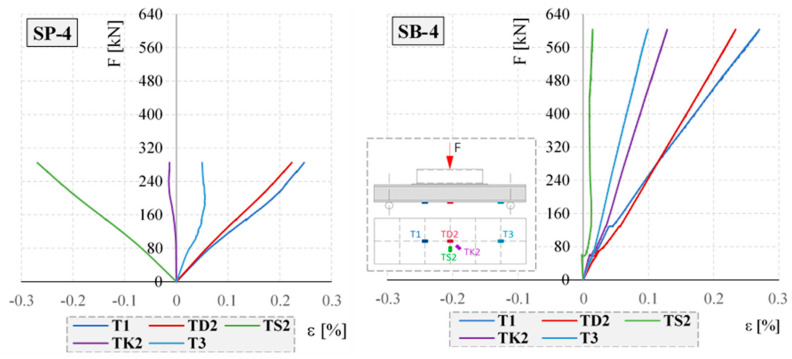
Strain–force relationships obtained from RFSGs for the SP-4 and SB-4 elements.

The strain–force relationships obtained from RFSGs for the selected “closed profiles” SP-4 and SB-4 are shown in Figure 17.

Analogous strain–force relationships were observed for all closed profiles tested. For the empty closed profiles (SP-1, SP-2, SP-3, and SP-4), strain gauges T1 and TD2 always showed the maximum longitudinal tensile strain, while strain gauge TS2 showed the maximum transverse compressive strain. Negative transverse strains in the TS2 gauges are the result of local deformation of the bottom surface of empty closed profiles. In contrast, in the concrete-filled elements, only the element with the smallest cross-section, SB-1, was measured with a negative transverse strain. In the other elements (SB-2, SB-3, and SB-4), the TS2 strain gauges indicated tensile strain.

The maximum tensile strains in the empty closed profiles were observed in elements SP-1 and SP-2 and were as follows: 0.36% and 0.38%. In the concrete-filled elements, the measured strain was up to 1.24% for element SB-1. The maximum compressive strains were measured in elements SP-2 and SP-3 and were 0.54% and 0.51%, respectively.

**Figure 18:**
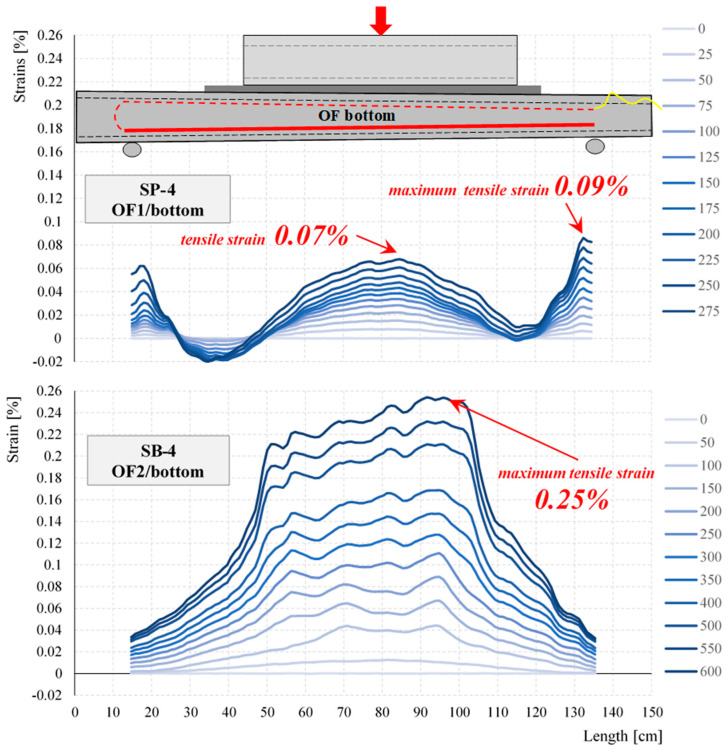
Strain distributions along the length of the bottom optical fibers at particular loading steps for SP-4 and SB-4.

**Figure 19:**
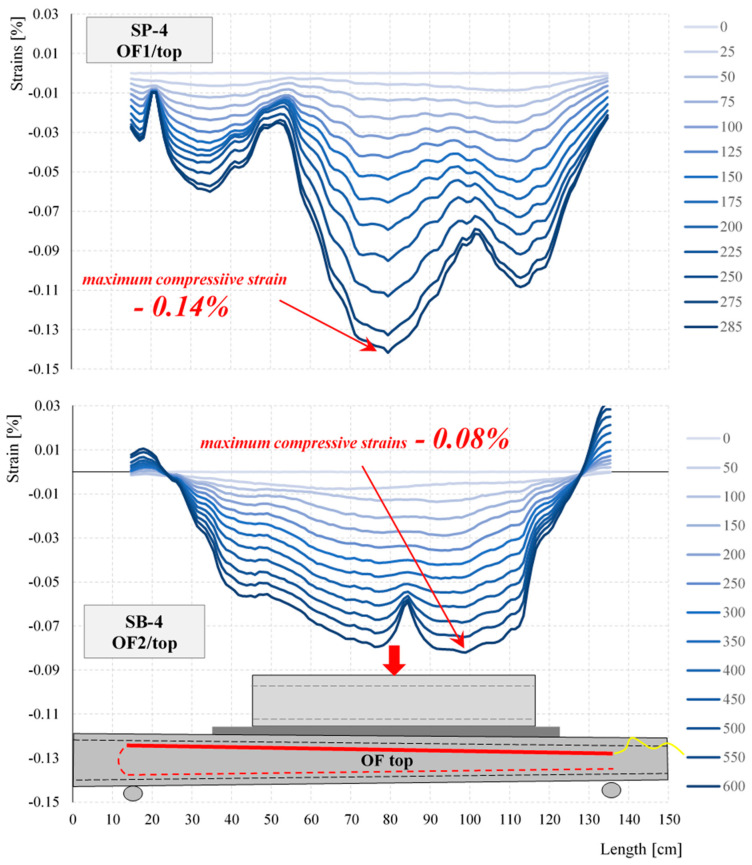
Strain distributions along the length of the top optical fibers at particular loading steps for SP-4 and SB-4.

The strain distributions registered during the selected stage of the test for selected elements of the same cross-section—empty SP-4 and concrete-filled SB-4—obtained from the optical fibers (OFs) are shown in Figure 18 and Figure 19. The asymmetry of the strain distributions shown in Figure 18 and Figure 19 is due to the variable cross-section of the test closed profiles. The marked lack of symmetry observed during the tests along the bottom and top lines of the strain distributions shown in Figure 18 and Figure 19 is an indication of where damage is most likely to occur. Local variations in the strain distribution (local minima) observed in the hollow elements are caused by the curvature of the upper surface of the profile and the change in the load pattern during the tests. As a result of the deformation of the profile, the load pattern changes from initially linear to two-point.

By analyzing the measured strains for all closed profiles, it was seen that despite the different failure loads, the maximum deformations for SP-1, SP-2, and SP-3 were similar. For example, the maximum tensile strains ranged from 0.15% to 0.24% for optical fiber measurements and from 0.25% to 0.31% for strain gauge T1 measurements. The same was true for strains in the compressed zone, which ranged from −0.22% to −0.27% for optical fiber measurements. In contrast, the strain values obtained for the SP-4 element were lower than those for the other empty closed profiles. Furthermore, due to it having the largest cross-sectional area of the profile and wall thickness, the highest failure load value was expected for the SP-4 profile. During the research, it became apparent that a higher destructive force was obtained for the SP-3 element. In the case of concrete-filled elements, it was observed that with increasing dimensions of the cross-sectional elements, the measured strains decreased. In the elements filled with concrete, SB-1, SB-2, SB-3, and SB-4, at a force of 600 kN, the tests were stopped for technical reasons (it was not possible to apply a higher load). The concrete-filled profiles did not fail.

**Figure 20:**
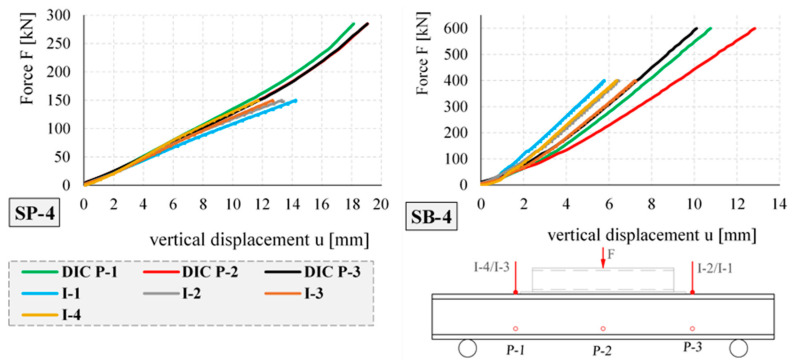
Displacements of tested elements SP-4 and SB-4.

The displacement–force relationships for selected elements of the same cross-section—empty SP-4 and concrete-filled SB-4—obtained during the tests are shown in Figure 20. Analogous to the boards, the displacements were measured using inductive sensors and the DIC system.

The maximum displacements of the SP-1, SP-2, SP-3, SP-4, SB-1, SB-2, SB-3, and SB-4 elements, measured by the DIC system for selected load steps and selected markers P-1, P-2, and P-3, are summarized in Table 5.

By analyzing the results in Table 5, it can be seen that the maximum measured displacements of all tested empty closed profiles (SP-1, SP-2, SP-3, and SP-4) were very similar, ranging from 18 to 23 mm. For the SB-1, SB-2, SB-3, and SB-4 concrete-filled elements, the displacements for a maximum force of 600 kN correlated with the stiffness of the tested elements. The largest displacement of 44 mm was measured for the SB-1 element with the smallest cross-section, and the smallest displacement of 13 mm was measured for the SB-4 element with the largest cross-section.

**Figure 21:**
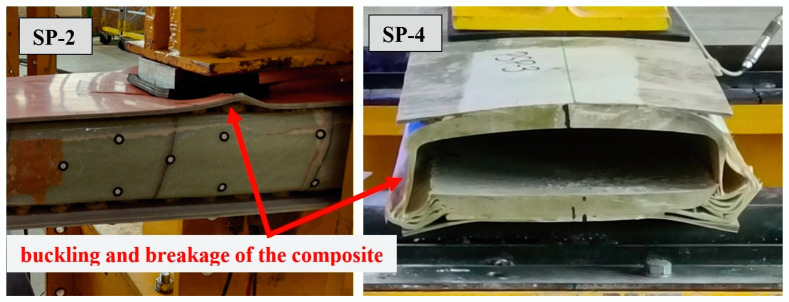
Failure mechanism of the empty closed profiles.

Two failure mechanisms were observed in the empty closed profiles. In elements SP-1 and SP-2, the failure was caused by the buckling of the compression chord of the box section. The authors of the publication [27] obtained an analogous failure mechanism for composite boxes of wind turbine blades. For elements SP-3 and SP-4, on the other hand, the failure was caused by delamination of the bottom chord at the support—as shown in Figure 21.

### 4.3. DFOS vs. DIC—Comparison of Component Deflection Line

Based on the strain profiles along the whole length, it is possible to determine the displacement of an analyzed section using the “trapezoidal method” [30]. This algorithm does not require knowledge of the properties of the material (e.g., elasticity module). It is based solely on the results obtained from virtual interconnected meters. The length of these meters is equal to the defined spatial resolution [10 mm], while the height is the distance between the optical fibers. A detailed description of the methodology is given in [30] and the general equation can be written as follows:

u = f(ε_b_, ε_t_, h_f_, l_f_, b_c_), (1)

where u—displacement of the measuring section [mm]; ε_b_—strain of the top optical fiber of the measuring section [μ_ε_]; ε_b_—strain of the bottom optical fiber of the measuring section [μ_ε_]; h_f_—distance between optical fibers [mm]; l_f_—spatial resolution [mm]; b_c_—boundary conditions (e.g., initial displacement and rotation angle or displacements in two known locations).

**Figure 22:**
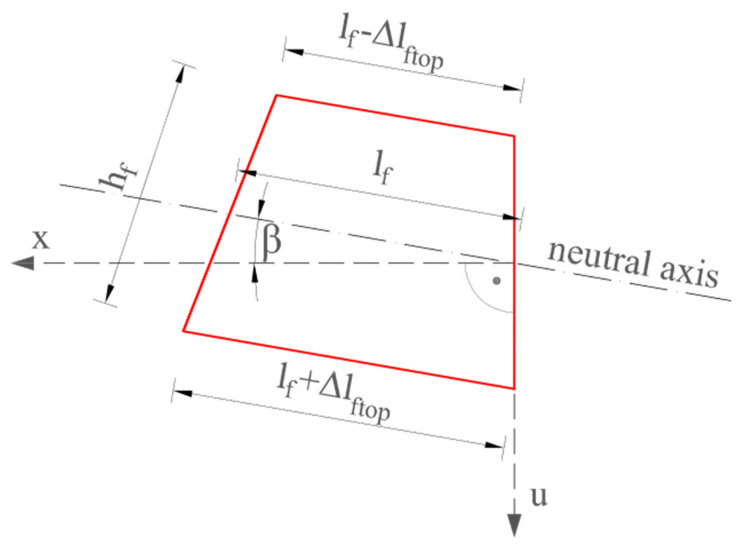
The concept of the trapezoidal method.

The trapezoidal method is based on the analysis of the deformation of a single trapezoidal, defined by the spatial resolution and distance between upper and lower optical fibers—Figure 22.

Once the displacement is calculated within the individual trapezoids, move to the next one and add the displacement value.

**Figure 23:**
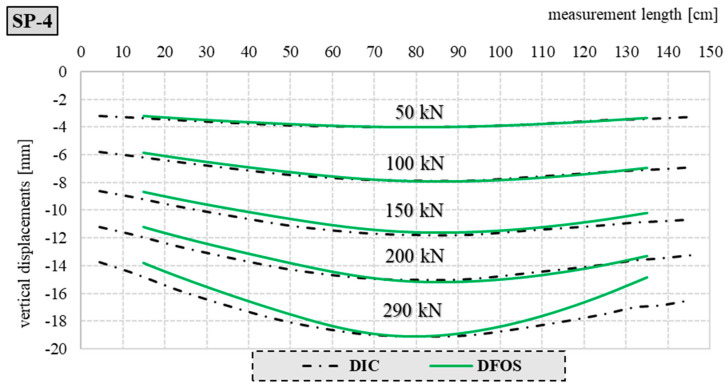
Comparison of deflection lines for the empty closed profile SP-4 and selected load steps.

**Figure 24:**
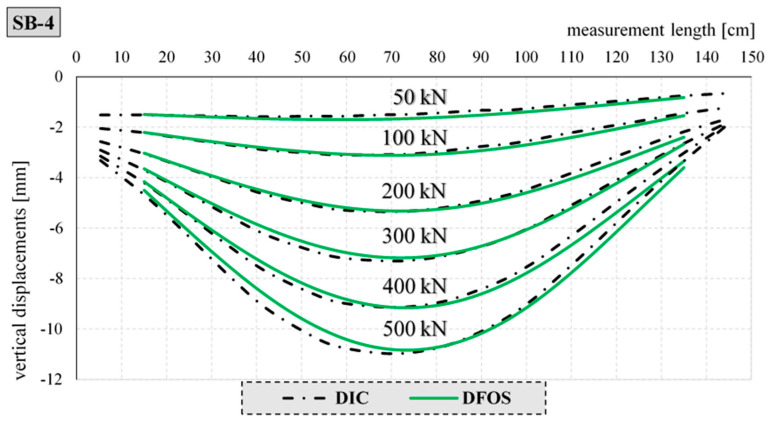
Comparison of deflection lines for the concrete-filled closed profile SB-4 and selected load steps.

Figure 23 and Figure 24 show a comparison of the deflection lines of the closed profiles obtained from the DIC and DFOS measurements. The deflection lines shown in the figures were determined for the axis of the element. The boundary conditions, i.e., the angle of rotation on the support and the vertical displacements required to determine the deflection line according to Formula (1), were derived from the DIC measurement technique.

Figure 23 shows a comparison of the deflection line for the SP-4 element (closed empty profile) and selected load steps.

Similarly, Figure 24 shows a comparison of the deflection line obtained by the DIC and DFOS methods for the selected element filled with concrete, SB-4.

The maximum difference in the measured values for both methods did not exceed 1 mm. In the case of elements filled with concrete, greater compliance was achieved than for the empty elements. This is due to the boundary conditions and the different angles of rotation of the empty elements over the supports.

### 4.4. Comparison of Results for Empty and Concrete-Filled Closed Profiles

**Figure 25:**
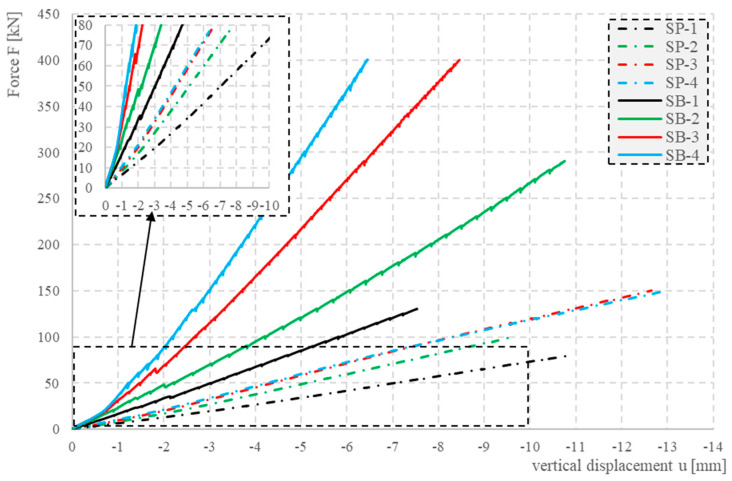
Load–displacement relationships for all the closed profiles.

Figure 25 shows the load–displacement relationships for all the closed profiles obtained from the inductive sensors.

The horizontal axis shows the averaged displacement values obtained from the four sensors arranged according to Figure 15. The vertical axis shows the load realized. Since, for safety reasons, the inductive sensors were removed during the tests, the load range presented is limited. The relationship shown in the figure is linear, typical of elements in the elastic range. Using this fact, the bending stiffness of the section under consideration can be determined from the measured deflections from Equation (2):

(2) $$u={{ʃ}_{l}\;M}_{1}\frac{M}{B}dx$$

where *u*—beam deflection; *M*_1_—bending moment value caused by the unit virtual force; *M*—bending moment value caused by the external loads; *l*—span length; *B*—stiffness of section.

Taking the bending moment calculated at the center of the span as *M*, the lowest bending stiffness of the element *B*_min_ can be determined. On the basis of the calculations carried out, it can be concluded that the stiffness of the concrete-filled elements is 2.3 to 3.3 times greater than that of the hollow elements.

Concrete-filled elements are characterized by significantly higher load-bearing capacities. Destruction of the hollow element with the smallest cross-section, SP-1, occurred at a force of 114 kN, while for the concrete-filled element, a load of 600 kN was achieved. The strain distributions of the concrete-filled and empty elements shown in Figure 18 and Figure 19 also differ. In the hollow members, the largest tensile deformations were observed at the supports, while in the filled members, they were observed in the span. Additionally, the strain distributions show that the empty elements were highly sensitive to local concentrated loads (kinks in the strain distributions at the point of concentrated forces; see Figure 18 and Figure 19). This sensitivity was not seen in the filled elements.

### 4.5. Summary

The presented research results indicate the possibility of using waste wind turbine blades for the construction of retaining walls while highlighting the advantages and disadvantages of such a solution. The research confirmed the authors’ initial assumption that fragments of LM-type blades could be used as filling elements and closed profiles filled with concrete from Vestas-type blades could be used as the main load-bearing elements, or “columns”. The results in Figure 17, Figure 18, Figure 19, Figure 20 and Figure 23, Figure 24, Figure 25 show a significant increase in the stiffness of the filled elements compared to the empty ones. Furthermore, the obvious benefit of the increasing cross-sectional area of the filled elements is useful to exploit by appropriate positioning on the cantilevered retaining wall. The results in Table 3 show a very strong influence of the thickness of the LM-type blade fragment used. As with the “columns”, the “boards” of LM type in the retaining wall should be placed in accordance with the increase in soil pressure, thus allowing them to be used efficiently.

A major difficulty in carrying out the research was the irregular shape of the fragments extracted from wind turbine blades and the significant variation in their physical parameters, which is a characteristic feature of glass fiber composite components. The irregular shapes can be exploited effectively by matching the elements to the internal forces of the retaining wall. However, problems in connecting these elements to each other cannot be avoided because of their shape. The high variability of the physical parameters forces a design with a correspondingly high safety factor, particularly as the tests carried out showed the variable and difficult-to-predict nature of element failure (Figure 16 and Figure 21).

## 5. Conclusions

This paper describes the material and structural testing of individual components obtained from used LM- and Vestas-type wind turbine blades for reuse as slope and trench reinforcement components.

A detailed analysis of the strength and deformability of blade components based on several reference techniques was the subject of this article. High agreement was observed between the results obtained from foil strain gauges and fiber optic sensors when comparing the measured strains of composite boards extracted from LM-type wind turbine blades. The maximum measured strains for a board thickness of 20 mm ranged from 1.04 to 1.25%, depending on the component, while for a board thickness of 30 mm, they ranged from 1.64 to 1.78%. In contrast to the measured strains, the failure forces in the tested boards were characterized by considerable variability. For the 20 mm thick boards, they were 20 kN and 42 kN, while for the 30 mm boards, they were 186 kN and 195 kN. The measured maximum displacements for the three elements (PD-1, PD-2, and PD-3) were very close, ranging from 200 to 233 mm, while much lower values were recorded for the PD-4 board. According to the research, there is a high degree of variability in the strength parameters and deformation in the composite planks made from LM-type wind turbine blades due to the various failure mechanisms.

In the case of closed profiles extracted from Vestas-type blades, the test results obtained for the concrete-filled elements are very promising. These elements are characterized by high strength and low deformation. The observed stiffness of concrete-filled elements compared to empty ones was more than twice as high. On the contrary, the increase in load capacity relative to the weakest elements (SP-4 and SB-4) was more than five times greater. The measured displacements and strains correlated to the cross-sections of the individual concrete-filled elements showed deflections ranging from 10 to 44 mm, while tensile strains measured by optical fibers ranged from 0.24% to 0.76%.

It is evident from the comparison of the measurement methods that the new DFOS and DIC measurement techniques provide more information. For the closed profile elements under study, which were characterized by cross-sectional variability, this was particularly important. The strain distributions along the lengths of the bottom and top of the elements, obtained from the optical fibers, were characterized by a different shape and a lack of symmetry, which made it possible to predict the location of the damage during the measurements. In the case of only RFSGs, such a precise analysis would be impossible. As the study has shown, the use of non-contact measuring techniques is very important in the case of components characterized by sudden brittle failure, such as wind turbine blades. Measurement of deflection up to failure was not possible in the tests due to damage to the inductive sensors. This could be achieved by using the DIC system, a non-contact measuring technique.

The information on the stiffness of the hollow and concrete-filled composite elements, the failure forces, the deformation behavior, and the failure mode obtained from the various measurement techniques used will allow future validation of numerical models for the design of slope protection walls and excavations using used wind turbine blades.

## Figures and Tables

**Table 1:** Dimensions of the Vestas profile samples.

	Dimensions of the Vestas Profile Samples							
[mm]	SP-1/SB-1		SP-2/SB-2		SP-3/SB-3		SP-4/SB-4	
	End	Front	End	Front	End	Front	End	Front
l (length)	1500							
h (height)	110	130	130	150	150	180	180	210
w (width)	245	280	280	315	315	345	345	400
t1 (thickness)	8	8	8	8	6	6	6	9
t2 (thickness)	7	12	12	19	19	22	22	25

**Table 2:** Average compressive strength (6 samples), flexural tensile strength (3 samples), and modulus of elasticity of concrete (3 samples) for filling Vestas profiles.

Compressive Strength		Tensile Strength		Modulus of Elasticity	
Average	Standard Deviation	Average	Standard Deviation	Average	Standard Deviation
$f_{cm}$ [MPa]	σ [MPa]	$f_{cm}^{flex}$ [MPa]	σ [MPa]	E_cm_ [MPa]	σ [MPa]
35.1	0.49	4.03	0.35	36.4	1.99

**Table 3:** Strain value at the mid-span of the elements obtained from RFSGs and in the center of the optical fibers (OFs).

Yielding Strains of the Strain Gauge [%]			Particular Loading Steps [kN]						
			Max F PD-4	Max F PD-3	F	F	F	Max F PD-1	Max F PD-2
			20	42	50	100	156	186	195
Boards	PD-1 th. 30 mm	T1	0.12	0.27	0.33	0.82	1.40	1.65	
		TD2	0.14	0.31	0.37	0.88	1.44	1.64	
		OF	0.15	0.33	0.40	0.93	1.61	-	
	PD-2 th. 30 mm	T1	0.11	0.25	0.31	0.74	1.38	1.78	-
		TD2	0.15	0.32	0.38	-	-	-	-
		OF	0.16	0.34	0.41	0.92	1.53	-	-
	PD-3 th. 20 mm	T1	0.21	-					
		TD2	0.41	-					
		OF	0.73	1.25					
	PD-4 th. 20 mm	T1	0.85						
		TD2	0.50						
		OF	1.04						
-	No result due to optical fiber or RFSGs failure								
	Load greater than the breaking load on the component								

**Table 4:** Displacement of the points P-1, P-2, and P-3 obtained for selected load steps of the composite board measured by the DIC system.

Displacement (u) [mm]			Particular Loading Steps [kN]						
			Max F PD-4	Max F PD-3	F	F	F	Max F PD-1	Max F PD-2
			20	42	50	100	156	186	195
Boards	PD-1 th. 30 mm	P-1	10	21	26	70	137	161	
		P-2	11	25	31	87	169	200	
		P-3	9	20	25	68	134	179	
	PD-2 th. 30 mm	P-1	9	19	23	62	125	160	166
		P-2	11	25	30	81	161	206	216
		P-3	7	16	20	56	110	149	156
	PD-3 th. 20 mm	P-1	94	167					
		P-2	134	233					
		P-3	100	179					
	PD-4 th. 20 mm	P-1	116						
		P-2	155						
		P-3	123						

**Table 5:** Displacement of the points P-1, P-2, and P-3 obtained for selected load steps of closed profiles.

**Deflection [u] of** **the Selected DIC Markers [mm]**			**Particular Loading Steps [kN]**				
			**Max F SP-1**	**Max F SP-2**	**max F SP-4**	**Max F SP-3**	**Max F SB-1, SB-2,** **SB-3, SB-4**
			**114**	**254**	**285**	**305**	**600**
Columns not filled with concrete	SP-1	P-1	18				
		P-2	-				
		P-3	14				
	SP-2	P-1	12	21			
		P-2	12	23			
		P-3	11	20			
	SP-3	P-1	9	18	19	20	
		P-2	9	19	21	22	
		P-3	8	17	18	19	
	SP-4	P-1	9	17	18		
		P-2	9	18	19		
		P-3	9	18	19		
Columns filled with concrete	SB-1	P-1	7	16	18	20	38
		P-2	8	19	22	23	44
		P-3	6	14	16	81	31
	SB-2	P-1	7	13	14	15	26
		P-2	8	15	16	18	31
		P-3	7	12	13	14	24
	SB-3	P-1	4	7	8	8	15
		P-2	4	8	9	9	17
		P-3	3	6	6	7	12
	SB-4	P-1	3	6	6	6	11
		P-2	4	6	7	8	13
		P-3	3	5	6	6	10

## Data Availability

The raw data supporting the conclusions of this article will be made available by the authors on request.

## References

[1] Recommendations for Policymakers: Research and Innovation Focus Composite Recycling Technologies of Existing Blades. https://etipwind.eu/files/reports/ETIPWind-How-wind-is-going-circular-blade-recycling.pdf.

[2] Liu P., Barlow C.Y. (2017). Wind turbine blade waste in 2050. Waste Manag..

[3] Jensen J., Skelton K. (2018). Wind turbine blade recycling: Experiences, challenges and possibilities in a circular economy. Renew. Sustain. Energy Rev..

[4] Khalid M.Y., Arif Z.U., Hossain M., Umer R. (2023). Recycling of wind turbine blades through modern recycling technologies: A road to zero waste. Renew. Energy Focus.

[5] Gentry T.R., Al-Haddad T., Bank L.C., Arias F.R., Nagle A., Leahy P. (2020). Structural Analysis of a Roof Extracted from a Wind Turbine Blade. J. Archit. Eng..

[6] Hasheminezhad A., Nazari Z., Yang B., Ceylan H., Kim S. (2024). A comprehensive review of sustainable solutions for reusing wind turbine blade waste materials. J. Environ. Manag..

[7] Kavaliauskas Ž., Kėželis R., Grigaitienė V., Marcinauskas L., Milieška M., Valinčius V., Uscila R., Snapkauskienė V., Gimžauskaitė D., Baltušnikas A. (2023). Recycling of Wind Turbine Blades into Microfiber Using Plasma Technology. Materials.

[8] Alshannaq A.A., Bank L.C., Scott D.W., Gentry R. (2021). A Decommissioned Wind Blade as a Second-Life Construction Material for a Transmission Pole. Constr. Mater..

[9] Alshannaq A., Scott D., Bank L., Bermek M., Gentry R. Structural Re-Use of De-Commissioned Wind Turbine Blades in Civil Engineering Applications. Proceedings of the 34th Technical Conference of American Society for Composites.

[10] André A., Kullberg J., Nygren D., Mattsson C., Nedev G., Haghani R. (2020). Re-use of wind turbine blade for construction and infrastructure applications. IOP Conference Series: Materials Science and Engineering.

[11] Deeney P., Nagle A.J., Gough F., Lemmertz H., Delaney E.L., McKinley J.M., Graham C., Leahy P.G., Dunphy N.P., Mullally G. (2021). End-of-Life alternatives for wind turbine blades: Sustainability Indices based on the UN sustainable development goals. Resour. Conserv. Recycl..

[12] Ruane K., Soutsos M., Huynh A., Zhang Z., Nagle A., McDonald K., Gentry T.R., Leahy P., Bank L.C. (2023). Construction and Cost Analysis of BladeBridges Made from Decommissioned FRP Wind Turbine Blades. Sustainability.

[13] Bank L.C., Gentry R., Leahy P. (2022). Re-Wind Design Catalog 2nd Edition Fall/Autumn 2022.

[14] Adamcio A. Anmet. https://www.anmet.com.pl/.

[15] Debnath A., Pal S.K. (2023). Influence of surcharge strip loads on the behavior of cantilever sheet pile walls: A numerical study. J. Eng. Res..

[16] Singh A., Chatterjee K. (2020). Lateral earth pressure and bending moment on sheet pile walls due to uniform surcharge. Geomech. Eng..

[17] Buda-Ożóg L., Zięba J., Sieńkowska K., Nykiel D., Zuziak K., Sieńko R. (2022). Bednarski Distributed fibre optic sensing: Reinforcement yielding strains and crack detection in concrete slab during column failure simulation. Measurement.

[18] Liu Y., Bao Y. (2023). Automatic interpretation of strain distributions measured from distributed fiber optic sensors for crack monitoring. Measurement.

[19] López-Higuera J.M., Cobo L.R., Incera A.Q., Cobo A. (2011). Fiber optic sensors in structural health monitoring. J. Light. Technol..

[20] Kopec M., Garbacz G., Brodecki A., Kowalewski Z.L. (2022). Metric entropy and digital image correlation in deformation dynamics analysis of fibre glass reinforced composite under uniaxial tension. Measurement.

[21] Wang J., Wang C., Ji Y., Qie R., Wang D., Liu G. (2024). Mechanical Properties and Microscopic Study of Recycled Fibre Concrete Based on Wind Turbine Blades. Materials.

[22] Zhen T., Zhao P., Zhang X., Si W., Ling T. (2023). The Effect of GFRP Powder on the High and Low-Temperature Properties of Asphalt Mastic. Materials.

[23] Pławecka K., Przybyła J., Korniejenko K., Lin W.T., Cheng A., Łach M. (2021). Recycling of mechanically ground wind turbine blades as filler in geopolymer composite. Materials.

[24] Alshannaq A.A., Respert J.A. Large-scale testing of a GFRP power transmission pole prototype made from a decommissioned GE37 wind turbine blade. Proceedings of the 11th International Conference on FRP Composites in Civil Engineering.

[25] Broniewicz M., Halicka A., Buda-Ożóg L., Broniewicz F., Nykiel D., Jabłoński Ł. (2024). The Use of Wind Turbine Blades to Build Road Noise Barriers as an Example of a Circular Economy Model. Materials.

[26] Alshannaq A.A., Respert J.A., Bank L.C., Scott D.W., Gentry T.R. (2022). As-Received Physical and Mechanical Properties of the Spar Cap of a GE37 Decommissioned Glass FRP Wind Turbine Blade. J. Mater. Civ. Eng..

[27] Ahmed M.M.Z., Alzahrani B., Jouini N., Hessien M.M., Ataya S. (2021). The role of orientation and temperature on the mechanical properties of a 20 years old wind turbine blade GFR composite. Polymers.

[28] Sayer F., Bürkner F., Buchholz B., Strobel M., van Wingerde A.M., Busmann H., Seifert H. (2013). Influence of a Wind Turbine Service Life on the Mechanical Properties of the Material and the Blade.

[29] Tang J., Chen X. (2018). Experimental investigation on ultimate strength and failure response of composite box beams used in wind turbine blades. Compos. Struct..

[30] Bednarski Ł., Sieńko R., Grygierek M., Howiacki T. (2021). New Distributed Fibre Optic 3DSensor with Thermal Self-Compensation System: Design, Research and Field Proof Application Inside Geotechnical Structure. Sensors.

